# Pathway‐specific polygenic risk scores modify the association between amyloid and tau PET accumulation in patients with Alzheimer's disease

**DOI:** 10.1002/alz.71939

**Published:** 2026-10-27

**Authors:** Carolina Valentim, Jannis Denecke, Simon Frerich, Tobey J. Betthauser, Yuanxi Li, Reihaneh Ahmadi, Nicolai Franzmeier, Alzheimer's Disease Neuroimaging Initiative (ADNI), Rainer Malik, Michael Ewers

**Affiliations:** ^1^ Institute for Stroke and Dementia Research University Hospital Ludwig Maximilian University Munich Germany; ^2^ Wisconsin Alzheimer's Disease Research Center University of Wisconsin School of Medicine and Public Health Madison Wisconsin USA; ^3^ Department of Medicine University of Wisconsin School of Medicine and Public Health Madison Wisconsin USA; ^4^ Munich Cluster for Systems Neurology (SyNergy) Munich Germany; ^5^ Department of Psychiatry and Neurochemistry The Sahlgrenska Academy Institute of Neuroscience and Physiology, University of Gothenburg Mölndal and Gothenburg Sweden; ^6^ German Center for Neurodegenerative Diseases (DZNE) Munich Germany

**Keywords:** Alzheimer's disease, amyloid chronicity, pathway analysis, polygenic risk score, positron emission tomography imaging

## Abstract

**BACKGROUND:**

Genome‐wide association studies (GWASs) identified multiple genetic variants associated with Alzheimer's disease (AD) risk, yet the biological pathways linking these to amyloid and tau pathology remain unclear.

**METHODS:**

We conducted a meta‐analysis of recent GWASs to identify independent genome‐wide significant single nucleotide polymorphisms which were mapped via gene‐set enrichment to derive pathway‐specific polygenic risk scores (pPRSs). Analyses included 441 amyloid‐positive participants from the Alzheimer's Disease Neuroimaging Initiative (ADNI; 224 with longitudinal tau positron emission tomography [PET]) and 250 amyloid‐positive participants from the Anti‐Amyloid Treatment in Asymptomatic Alzheimer's Disease (A4) study (113 with longitudinal tau PET). Robust linear regressions tested cross‐sectional and longitudinal pPRS‐by‐amyloid chronicity interactions on tau PET.

**RESULTS:**

Four pPRSs were identified including adaptive immune response, leukocyte activation, transport, and intracellular signaling. Only the transport pPRS strengthened amyloid–tau coupling cross‐sectionally across all Braak stages in ADNI (*p* < 0.05) and at Braak I in A4 (*p* < 0.05), with stronger effects observed in females; in ADNI, these associations persisted longitudinally only at the higher Braak stages.

**DISCUSSION:**

Genetic variation in transport pathways modulates amyloid‐driven tau accumulation, revealing mechanisms shaping AD progression, beyond apolipoprotein E.

## Highlights

- Vesicular trafficking and endosomal transport pathway‐specific polygenic risk score (pPRS) amplifies amyloid‐driven tau accumulation cross‐sectionally at Braak I in both the Alzheimer's Disease Neuroimaging Initiative (ADNI) and an independent preclinical cohort (Anti‐Amyloid Treatment in Asymptomatic Alzheimer's Disease [A4]), with the strongest effects at established cortical (Braak III–VI) stages in ADNI.
- Longitudinally in ADNI, the transport pPRS effect was present as a trend at Braak I and reached significance at higher Braak stages (III–VI); the corresponding longitudinal effect in A4 did not reach significance.
- The transport pPRS effect reflects the aggregate contribution of the pathway's trafficking machinery (e.g., *BIN1, PICALM, SORL1, SNX1, TOMM40*) rather than a small subset of high‐weight genes.
- In exploratory sex‐stratified analyses, the transport pathway–PRS effect on tau positron emission tomography was more pronounced in females than males in both cohorts.

## 1 BACKGROUND

Alzheimer's disease (AD) is characterized by the accumulation of amyloid beta (Aβ) and tau pathology, with tau pathology being more closely linked to the emergence of dementia symptoms. Longitudinal biomarker studies suggest that amyloid deposition precedes and increases the likelihood of subsequent tau accumulation, indicating that tau develops downstream of Aβ.^1^ However, even among individuals with comparable amyloid burden, tau deposition shows substantial heterogeneity,^2^ suggesting that additional factors modulate the relationship between Aβ and tau pathology in AD.

Large‐scale genome‐wide association studies (GWASs) have identified numerous common and rare variants that contribute to AD dementia risk.^3^, ^4^, ^5^, ^6^ The apolipoprotein E (*APOE*) ε4 allele, the strongest common genetic risk factor for AD, has been associated with greater amyloid‐related increase in pathologic tau.^5^, ^7^, ^8^ Beyond *APOE*, however, emerging evidence suggests that a broader polygenic architecture influences susceptibility to tau pathology. Higher global polygenic risk scores (PRSs), which combine GWAS‐derived AD risk single nucleotide polymorphisms (SNPs),^6^, ^7^ have been associated with higher cerebrospinal fluid (CSF) levels of phosphorylated tau and greater tau positron emission tomography (PET) uptake.^5^, ^8^, ^9^ In particular, a higher global PRS was associated with a steeper relationship between amyloid PET uptake and subsequent rate of tau PET accumulation rate, suggesting that polygenic risk may modulate biological pathways downstream of Aβ.^5^ Yet, because global PRSs aggregate a large number of SNPs across the genome, they do not allow for the identification of specific biological pathways that may drive tau development, nor do they address which pathways specifically modulate the dynamic relationship between Aβ deposition and tau accumulation.^10^, ^11^, ^12^

Pathway‐specific PRSs (pPRSs) quantify genetic risk attributable to genes within specific biological pathways and have been applied to test associations with AD risk and biomarkers, including tau pathology.^10^, ^13^, ^14^

Here, we used a clock model of amyloid accumulation^15^, ^16^ to examine how amyloid chronicity, defined as the time since Aβ first became abnormal, relates to tau PET accumulation. We then estimated pPRS based on SNPs derived from meta‐analysis of two of the largest recent AD GWASs^3^, ^4^ to determine which biological pathways modulate the association between amyloid chronicity and tau PET accumulation. Given evidence for sex‐dependent genetic susceptibility to pathologic tau,^17^, ^18^, ^19^ we additionally examined these pathway‐specific effects separately in men and women.

## 2 METHODS

### 2.1 Participants

#### 2.1.1 Alzheimer's Disease Neuroimaging Initiative cohort

We included 441 amyloid PET–positive participants (mean age: 75.1  ±  7.6 years) from the Alzheimer's Disease Neuroimaging Initiative (ADNI; phase II and III), a multicenter observational study designed to characterize longitudinal biomarker changes in aging and AD (for details, see http://adni.loni.usc.edu/).^20^ The sample comprised 202 cognitively unimpaired (CU) participants, 162 participants with mild cognitive impairment (MCI), and 77 participants with AD dementia.

**RESEARCH IN CONTEXT**

1. **Systematic review**: We searched PubMed for studies examining the genetic modulation of tau pathology in Alzheimer's disease (AD). While previous studies have assessed the association between mono‐ or polygenic risk on biomarkers of pathologic tau in AD, there is a research gap on which biological pathways are tapped by polygenic risk profiles that modulate the temporal association between amyloid and tau pathology during the course of AD.
2. **Interpretation**: Our findings demonstrate that genetic risk variants, which cluster primarily on genes involved in vesicular trafficking and endosomal transport, modulate amyloid‐related regional fibrillar tau: individuals with higher transport polygenic risk scores showed higher levels and higher rates of tau positron emission tomography increases during amyloid accumulation as measured by time since becoming first amyloid positive. This effect was detectable cross‐sectionally at the earliest affected region (Braak stage I) in both the discovery cohort (Alzheimer's Disease Neuroimaging Initiative [ADNI]) and an independent preclinical cohort (Anti‐Amyloid Treatment in Asymptomatic Alzheimer's Disease), and extended longitudinally to higher cortical stages (Braak III–VI) in ADNI. Exploratory analyses further suggested this effect was more pronounced in females than males in both cohorts. These findings suggest that cellular transport–related biological pathways may be involved in the development of tau pathology downstream of amyloid pathology, with a potential sex‐specific component.
3. **Future directions**: Amyloid chronicity clocks provide a principled framework for detecting stage‐dependent genetic effects that cross‐sectional designs miss. Future work should examine whether sex differences in amyloid precursor protein trafficking and endosomal–vesicular transport mechanistically explain the heightened tau vulnerability observed in women.

Inclusion criteria for the current study were the availability of genome‐wide genotype data, at least one [^1^ ^8^F]flortaucipir (AV‐1451) tau PET scan, and an amyloid PET scan ([^1^ ^8^F]florbetapir or [^1^ ^8^F]florbetaben) obtained within 30 days of the baseline tau PET scan. Amyloid PET positivity (Aβ+) was defined using ADNI PET Core thresholds (see https://tinyurl.com/3jjn7mu7; [^1^ ^8^F]florbetapir standardized uptake value ratio [SUVR] > 1.11 or [^1^ ^8^F]florbetaben SUVR > 1.08; see https://tinyurl.com/5yfe9rny). For longitudinal tau PET scans, the follow‐up tau PET scans were acquired approximately between 9 and 18 months apart. A flowchart of participant selection is shown in Figure S1 in supporting information.

Participants were classified according to ADNI diagnostic criteria as CU (Mini‐Mental State Examination [MMSE] ≥ 29, Clinical Dementia Rating = 0, no memory concerns), MCI (MMSE 24–28, memory deficits on the Wechsler Memory Scale II with preserved daily functioning), or AD dementia based on standard clinical criteria.^21^ The study was approved by institutional review boards at participating centers, and participants provided written informed consent in accordance with ADNI guidelines and ethical regulations.

#### 2.1.2 Anti‐Amyloid Treatment in Asymptomatic Alzheimer's Disease cohort

We included 250 amyloid PET–positive participants from the Anti‐Amyloid Treatment in Asymptomatic Alzheimer's Disease (A4) study, which enrolled cognitively normal, community‐dwelling older adults aged 65 to 85 years with biomarker evidence of elevated amyloid on PET (https://www.a4studydata.org). Amyloid PET–positive participants were randomized to placebo or solanezumab treatment arms in a clinical trial.^22^ For the present study, which included longitudinal tau PET, we restricted the sample to amyloid PET–positive participants who were randomized to the placebo arm (*n* = 591), using in addition the following inclusion criteria: (1) availability of genome‐wide genotype data, (2) at least two longitudinal [^1^ ^8^F]flortaucipir (AV‐1451) tau PET scans acquired ≈ 12 months apart, and (3) an amyloid PET scan confirming amyloid positivity using a Centiloid threshold of 21. A full overview of participant selection is provided in Figure S1. All study protocols were approved by institutional review boards at participating centers, and all participants provided written informed consent in accordance with A4 study guidelines and ethical regulations.

### 2.2 Genotyping procedures and quality control

#### 2.2.1 ADNI cohort

Genotype data were obtained from the ADNI database (https://adni.loni.usc.edu). Genotyping was conducted using three Illumina arrays: Human610‐Quad (620,901 variants), HumanOmniExpress (730,525 variants), and HumanOmni2.5‐8 (2,379,855 variants). Missing genotypes were imputed using the Haplotype Reference Consortium (HRC) reference panel v1.1 via Minimac4 on the TOPMed Imputation Server.^23^ Imputations were performed separately for each cohort (ADNI‐2 and ADNI‐3) due to the use of different genotyping arrays. Pre‐imputation processing included updating strand orientation, genomic positions, and reference/alternative allele assignments to ensure consistency with the HRC reference panel.

SNPs were excluded in case of inconsistent alleles, allele frequency differences > 0.2, absence of equivalent allele in the reference panel, or being palindromic SNPs with a minor allele frequency (MAF) > 0.4 (via https://www.chg.ox.ac.uk/~wrayner/tools/, HRC‐1000G Version 4.3.0). Quality control (QC) also involved removing SNPs with imputation *R* ^2^ < 0.5. Principal components (PCs) for correction of population stratification were computed from the QC'd genotyped SNPs after a minor allele frequency filter (MAF ≥ 0.05), linkage disequilibrium (LD) pruning (PLINK–indep‐pairwise 200 50 0.1; 200‐variant window, 50‐variant step, *r* ^2^ threshold 0.1), and removal of related individuals (KING kinship < 0.0884, i.e., closer than second degree), using PLINK 2.0–pca; the first five components (PC1–PC5) were retained as covariates in downstream statistical models.

#### 2.2.2 A4 cohort

Genotype data were obtained from the A4 database (https://www.a4studydata.org). The A4 study obtained genotyped data from 3465 participants genotyped on the Illumina Global Screening Array, yielding 700,078 variants on genome GRCh37. HRC imputation preparation was made by the A4 study group using scripts from the McCarthy Group (https://www.well.ox.ac.uk/~wrayner/tools/, Version 4.2.10). Standard pre‐imputation QC excluded variants with genotyping efficiency < 95%, MAF < 1%, or Hardy–Weinberg equilibrium *p* < 1 × 10^−^^6^, as well as samples with call rates < 99%, sex discordance, excess relatedness (pi‐hat > 0.25), or outlying heterozygosity (> 5 standard deviation [SD]). PCs were calculated using 1000 Genomes Project Phase 3 European reference panel,^24^ and ancestry outliers (> 5 SD from the non‐Hispanic White mean) were removed. Imputation was performed on the TOPMed Imputation Server^23^ using Minimac4 and Eagle for phasing, after harmonization of variant positions, strand orientations, and reference/alternative allele assignments against the TOPMed^23^ reference panel (GRCh38). Palindromic, multi‐allelic, and duplicated variants were excluded prior to imputation. After filtering for imputation quality (*R* ^2^ > 0.8) and removal of multiallelic SNPs, the final imputed dataset comprised 3008 non‐Hispanic White individuals and 35,276,281 variants. For A4, PCs were calculated using the 1000 Genomes Project Phase 3 European reference panel,^24^ and ancestry outliers (> 5 SD from the non‐Hispanic White mean) were removed; the first five components (PC1–PC5) from this analysis were retained as covariates in downstream statistical models.

### 2.3 GWAS meta‐analysis for variant selection

To identify those variants that were significantly associated with AD and related dementias, we conducted a fixed‐effects meta‐analysis using METAL,^25^ applied to the genome‐wide summary statistics from Bellenguez et al.^4^ and Wightman et al.^3^ Genome‐wide summary statistics from Bellenguez et al.^4^ were obtained from the GWAS Catalog (accession ID: GCST90027158) and summary statistics for Wightman et al.^3^ were obtained from publicly available consortium resources (PGCALZ2ExcludingUKBand23andME_METALInverseVariance_MetaAnalysis), corresponding to the meta‐analysis described in the original publication.

The resulting meta‐analysis summary statistics were processed using PLINK2^26^,^27^ to identify independent genome‐wide significant loci. Clumping was performed using the 1000 Genomes Project Phase 3 European reference panel,^24^ applying a significance threshold of *p* ≤ 5×10^−^^8^, an LD threshold of *r* ^2^ < 0.1, and a clumping window of 250 kb. This procedure yielded 321 independent genome‐wide significant SNPs, with a substantial fraction mapping to the *APOE* locus.

A final set of 291 independent significant SNPs remained after QC steps including filtering on MAF ≥ 0.01, harmonization with dbSNP‐build‐151 (GRCh37) identifiers, resolution of strand flips and multiallelic sites, and alignment to cohort‐specific genotype formats (ADNI, A4). These curated, independent genome‐wide significant variants were carried forward into subsequent PRS analyses.

Of 291 clumped lead SNPs, 37 were removed (including the *APOE* variants rs429358 and rs7412 for PRS construction, and variants mapping to the immunoglobulin heavy‐chain [IGH] locus to avoid spurious enrichment), leaving 254 curated SNPs mapping to 212 genes. Variants of the broader *APOE* locus, defined as chr19:44.4‐45.5 Mb [GRCh37] were not excluded; their influence was examined in a sensitivity analysis (Supplementary Material in supporting information). SNP‐to‐gene assignment was then carried out using SNPXplorer^28^ with tissue context set to GTEx Whole Blood, selected due to its relevance to its systemic and shared cerebral processes implicated in AD.

Cohort‐specific harmonization was required prior to PRS computation. For ADNI, variant identifiers were matched directly using rsIDs on GRCh37. For A4, additional preprocessing steps were necessary because the TOPMed^23^‐imputed genotype data are on GRCh38 and use positional variant identifiers (chr:pos:ref:alt) rather than rsIDs. Variant positions were therefore lifted over from GRCh38 to GRCh37 using UCSC liftOver,^29^ unmapped variants were excluded, and the score file was adapted to use positional identifiers to ensure unambiguous variant matching. PLINK2^26^,^27^ scoring then retained only variants present and unambiguously allele aligned in each cohort, yielding 172 SNPs in ADNI (82 excluded due to coverage gaps and conservative allele matching, including palindromic and multi‐allelic cases) and 125 SNPs in A4 for PRS computation.

The smaller number of variants available in A4 reflects not only differences in identifier format and array coverage, but also the more stringent post‐imputation quality filter applied in A4 (*R* ^2^ > 0.8) relative to ADNI (*R* ^2^ > 0.5), which may have led to the exclusion of variants that were retained in ADNI. Missing variants in both cohorts were handled by mean imputation within plink2–score.^26^, ^27^

### 2.4 Gene‐to‐pathway mapping

Gene‐to‐pathway mapping was applied to the 254 curated independent genome‐wide significant SNPs obtained through clumping. The mapped genes were subsequently tested for enrichment with G:Profiler,^30^ focusing on Gene Ontology Biological Processes (GO:BP). Pathways meeting a significance threshold of *p* ≤ 0.01 were retained for downstream analysis.

To reduce redundancy among enriched GO terms and to allow subsequent clustering, a semantic similarity matrix was constructed based on the Lin similarity metric,^31^, ^32^, ^33^ adapted from Tesi et al.^28^, ^34^ Hierarchical clustering was performed using the Ward.D2 method^35^ followed by dynamic tree cutting to group semantically overlapping pathways into broader functional categories.^8^, ^28^ *APOE* was retained during mapping and clustering to preserve pathway structure but was removed prior to PRS computation. SNPs mapping to the IGH locus were excluded to avoid spurious enrichment.

### 2.5 Calculation of pPRS

Because some SNPs were associated with multiple clusters, which renders the SNP less specific for a given cluster, a weighted mapping factor was computed for each SNP. This mapping factor was defined as the proportion of a SNP's total pathway associations attributed to a given cluster (i.e., the number of times a SNP was mapped to that cluster divided by its total number of cluster assignments):

$$\;w_{i,c}=\frac{n_{i,c}}{\sum_{c^{\prime }}n_{i,c^{\prime }}}\;$$

where *w*(*i,c*) is the mapping factor for SNP *i* in cluster *c*, *n*(*i,c*) is the number of times SNP *i* was assigned to cluster *c*, and Σ *n*(*i,c*') is the total number of cluster assignments across all clusters for that SNP. A higher mapping factor indicated that a SNP showed a stronger and more specific association with that cluster compared to all others. Detailed pathway selection criteria, enrichment statistics, and clustering procedures are provided in a GitHub repository (https://github.com/c‐valenti/PathwayPRS_AD).

pPRSs were then calculated using PLINK2,^26^,^27^ as the weighted sum of effect allele dosages for pathway‐specific SNPs, with weights given by the beta‐coefficients derived from the meta‐analytic GWAS for each SNP and the pathway‐mapping factors.

This approach generated pPRS estimates for each subject, with the number of estimates corresponding to the four identified pathway clusters. Formally, for subject *i* and pathway cluster *p*, the PRS was defined as:

$$PR\;S_{p,i}=\sum_{k=1}^{m}\beta_{k}\cdot \;M_{p,k}\cdot \;G_{i,k}$$

where *M* = 254, the total number of SNPs considered across all pathway clusters; β*_k_* is the GWAS effect size of SNP *k*, *M_p_,_k_* is the mapping factor of SNP *k* to pathway *p* (*M_p_,_k_* = 0 for SNPs with no mapping to pathway *p*, and *M_p_,_k_* summed across all four pathways equals 1 for each SNP); and *G_i_,_k_* is the effect allele dosage of SNP *k* for subject *i*. As individual SNPs could map to more than one pathway cluster, a given SNP may contribute, at fractional weight, to more than one pPRS. The final PRS for each pathway thus reflects both genetic effect size and functional specificity, excluding *APOE* variants to isolate non‐*APOE* effects. Full implementation details and PLINK2^26^, ^27^ scoring scripts are available in a GitHub repository (https://github.com/c‐valenti/PathwayPRS_AD).

### 2.6 Image acquisition and processing

All MRI data in the ADNI cohort were acquired on 3T MRI scanners using standardized protocols detailed in the ADNI database (https://adni.loni.usc.edu/data‐samples/adni‐data/neuroimaging/mri/). PET imaging was performed separately using dedicated PET scanners according to ADNI standard acquisition protocols.^36^ Tau PET scans were performed in six 5‐minute blocks, 75 to 105 minutes post‐injection of [^18^F] flortaucipir. Amyloid PET imaging was conducted in four 5‐minute blocks, either 50 to 70 minutes post‐injection of [^18^F]florbetapir or 90 to 110 minutes post‐injection of [^18^F]florbetaben. PET images were co‐registered, averaged, and standardized for orientation, voxel size, and intensity by the ADNI PET Core.

T1‐weighted (T1w) MRI images were processed using the CAT12 toolbox (see https://neuro‐jena.github.io/cat12‐help/). All images were screened for artifacts before preprocessing, and processing was conducted independently for the ADNI and A4 samples. Prior to MRI processing, dynamically acquired tau and amyloid PET images were first realigned and averaged to obtain single static images, and subsequently rigidly co‐registered to the participant's T1w images in native space. T1w images underwent bias field correction, brain extraction, and segmentation into gray matter, white matter, and CSF tissue maps using the CAT12 longitudinal cortical thickness pipeline. The preprocessed T1w images were then non‐linearly normalized to Montreal Neurological Institute (MNI) space using CAT12. The registration parameters obtained from this process were applied to transform the regions of interest (ROIs) of the MindBoggle DKT atlas^37^ from MNI space to individual PET scans in native space, masked with patient‐specific gray matter prior to SUVR extraction. Tau PET SUVRs were calculated by normalizing the SUVRs in each gray matter ROI to the mean uptake in the eroded inferior cerebellar gray matter, following the ADNI [^1^ ^8^F]flortaucipir (AV‐1451) processing protocol.^36^ For amyloid PET imaging, global SUVRs were determined by calculating the mean tracer uptake across cortical gray matter regions (frontal, lateral temporal, lateral parietal, and anterior/posterior cingulate) and normalizing to whole cerebellum uptake. Predefined SUVR cutoffs were used to define amyloid PET positivity, with thresholds set at > 1.11 for [^1^ ^8^F]florbetapir and > 1.08 for [^1^ ^8^F]florbetaben. To ensure comparability of amyloid PET data across different tracers, global SUVR values were subsequently converted the Centiloid scale^38^ using tracer‐specific equations provided by ADNI (for more information, see https://adni.loni.usc.edu/data‐samples/adni‐data/neuroimaging/pet/).

Composite tau PET values were computed for three composite ROIs corresponding to Braak *post mortem* staging of tau pathology.^39^ Standard Braak stage ROI definitions combine entorhinal cortex and hippocampus into a single transentorhinal (Braak I–II) stage. In the present analysis, we used Braak I (entorhinal cortex), excluding Braak II (hippocampus), to minimize the influence of non‐specific flortaucipir binding known to affect hippocampal signal. Braak III and IV (limbic) comprised inferior temporal, fusiform, parahippocampal, and amygdala regions; and Braak V and VI (isocortical) encompassed widespread association cortices including frontal, parietal, and occipital regions.

### 2.7 Amyloid chronicity modeling

We estimated amyloid chronicity as the estimated time elapsed since an individual's global amyloid PET levels, expressed in Centiloids, first reached the amyloid‐positivity threshold. To this end, we used sampled iterative local approximation (SILA),^15^ a data‐driven method for modeling longitudinal amyloid PET accumulation that estimates both the age of onset of amyloid abnormality and the time elapsed since that onset. Briefly, in the first step, iterative local smoothing of linear fits was applied to longitudinal Centiloid values from all ADNI participants for whom amyloid PET sessions were available (n = 1642; ADNI‐1; ADNI‐GO, ADNI‐2, ADNI‐3, and ADNI‐4), irrespective of tau‐PET availability, yielding a population‐level trajectory curve of amyloid accumulation over time (years). ADNI was selected as the reference cohort for trajectory estimation given its longitudinal design and broader range of amyloid burden, spanning sub‐threshold to advanced accumulation, whereas the A4 study by design enrolled only amyloid‐positive individuals. Amyloid chronicity for both ADNI and A4 participants was then estimated by projecting individual Centiloid values onto this ADNI‐derived trajectory curve, preserving the independence of the A4 sample. Amyloid positivity was defined using a standard Centiloid threshold of 21, as confirmed by Gaussian mixture modeling of the ADNI Centiloid distribution in the present sample.^38^, ^40^, ^41^, ^42^ Amyloid chronicity was then expressed as the time required for a given individual to reach this threshold and estimated age of onset of amyloid chronicity was calculated as the individual's current age minus amyloid chronicity (in years). For individuals who had not yet crossed the threshold, SILA estimated the number of years remaining until amyloid‐positivity status was predicted to be reached.

All SILA‐based estimations were performed using the MATLAB implementation provided (https://github.com/Betthauser‐Neuro‐Lab/SILA‐AD‐Biomarker; see also Figure S2 in supporting information).

### 2.8 Statistical analysis

The primary analyses tested whether pPRS modulated the association between amyloid chronicity and tau PET uptake at baseline. First, tau PET SUVR values for each of the three composite Braak stage ROIs were residualized against the first five genomic PCs (PC1–PC5, derived from directly genotyped SNPs after standard QC and LD pruning) by regressing the SUVR of each Braak stage composite onto the PCs. This step removed variance attributable to population stratification directly from the outcome. All subsequent primary, longitudinal, and sensitivity models used these PC‐residualized tau PET values as the dependent variable.

We next tested whether this relationship was modulated by pathway‐specific genetic risk. Robust linear regression models with Huber weighting were used to account for potential outliers and heteroscedasticity. Separate cross‐sectional models were estimated for each of the three PC‐residualized composite tau PET Braak stage ROIs, as the dependent variable, with the interaction of amyloid chronicity and pPRS as the main predictor. Models were adjusted for the interaction between amyloid chronicity and *APOE* ε4 carrier status (defined as presence of one or more ε4 alleles), pPRS, amyloid chronicity, age, sex, and years of education. In addition to the pathway‐specific analyses, we calculated a global PRS to evaluate whether the observed effects were specific to individual genetic pathways or instead reflected aggregate polygenic risk across the pathways. The global PRS was calculated using the same GWAS‐derived SNP effect‐size estimates and included all SNPs retained in the pathway‐clustering procedure across all clusters, without pathway‐based SNP partitioning. Thus, the global PRS represents the aggregate score across the complete clustered SNP set rather than a score restricted to any individual pathway. The interaction between global PRS and amyloid chronicity on tau PET was tested in regression analyses using the same covariates as included for the pathway‐specific analyses. Results from these analyses are reported in Table S1 in supporting information.

To examine whether baseline amyloid chronicity and pPRS predicted change in tau PET over time, we restricted the analyses to participants with longitudinal tau PET data (ADNI: *n* = 224; A4: *n* = 113). Subject‐level rate of change (ROC) values were computed for each Braak stage ROI using linear mixed‐effects models with random intercepts and slopes per participant, where the ROC in tau PET SUVR was modeled as a function of time (years) since the first tau PET scan. The resulting individual slopes of tau PET changes were residualized against PC1 to PC5 following the same procedure as the cross‐sectional outcomes, and the residuals were entered as dependent variables in robust regression models, and the interaction pPRS by amyloid chronicity, age, sex, years of education, and the interaction between amyloid chronicity and *APOE* ε4 carrier status as predictors.

Analyses were conducted in the A4 sample to assess the generalizability of findings from ADNI to a preclinical, cognitively unimpaired population, applying the same analytical framework, including PC residualization of tau PET outcomes, as in ADNI. Pathway‐specific PRSs were computed separately using the same GWAS‐derived beta coefficients, pathway mapping factors, and cluster structure, applied to each cohort's available genotype data as described above. Amyloid chronicity in A4 was estimated by projection onto the ADNI‐derived SILA trajectory curve rather than modeled independently, given that A4 enrolled only amyloid‐positive individuals. All remaining definitions and model structures were identical to those used in ADNI.

Sensitivity analyses examined the robustness of primary findings by excluding influential observations, identified using Cook distance applied independently to each linear regression model (fit on the PC‐residualized tau PET outcome) across Braak stage ROIs. Observations with a Cook distance exceeding the conventional threshold of 4/*n* were flagged as influential, where *n* denotes the number of observations contributing to that model. Applying this criterion, 59 influential observations were identified in ADNI cross‐sectional models (*n* = 382 remaining) and 31 in longitudinal rate‐of‐change models (*n* = 193 remaining), with per‐model counts varying by Braak region and PRS cluster. In A4, 26 influential observations were excluded in the cross‐sectional models (*n* = 224 remaining) and 14 in the longitudinal models (*n* = 99 remaining). In these reduced samples, we re‐tested the interaction between the transport pPRS and amyloid chronicity on PC‐residualized tau PET Braak stage ROI values using the same linear regression framework as in the primary analyses. Second, to determine whether the findings depended on the use of SILA‐derived amyloid chronicity, we repeated the primary analyses using amyloid PET Centiloid values in place of SILA‐derived amyloid chronicity across all four pathway‐specific PRS. The same model structure and covariates were retained. Lastly, to assess whether the observed associations were robust to inclusion of *APOE*‐related genetic variation, we recalculated all four pPRSs using the full SNP set, including *APOE* variants, and re‐fitted the primary cross‐sectional and longitudinal interaction models using the *APOE*‐inclusive scores in place of the original *APOE*–excluded scores.

Given the evidence for sex‐related differences in levels of tau pathology in AD,^17^, ^19^ we additionally performed exploratory sex‐specific analyses in both cohorts to test whether pPRS modulated amyloid‐related tau PET uptake differently in females compared to males. Propensity score (PS) matching was applied within each dataset to minimize baseline imbalances in demographic and clinical variables (age, education, amyloid burden, *APOE* ε4 carrier status, diagnosis, and pPRS). Matched samples included 169 females and 169 males in ADNI, and 86 females and 86 males in A4. Sex‐stratified robust regression models were then estimated, incorporating PS‐derived weights and including interaction terms between amyloid chronicity and pPRS. Additional exploratory analyses examined whether pathway‐specific genetic effects were associated with the spatial distribution of tau PET pathology. Data‐driven tau PET spatial subtypes were derived using the Subtype and Stage Inference (SuStaIn) algorithm.^43^, ^44^ We then tested whether pathway‐specific pPRS scores differed between the resulting tau PET spatial subtypes using analyses of covariance, adjusting for age, sex, education, *APOE* ε4 carrier status, and amyloid chronicity. Subtype assignments at different probability thresholds were examined to assess the robustness of the findings to classification confidence (Supplementary Methods in supporting information). In parallel, because previous findings suggested that tau PET can be distributed asymmetrically in the brain,^45^ we tested whether the pPRS x amyloid chronicity association differed between hemispheres by including a pPRS x amyloid chronicity x hemisphere interaction term.

In order to guard against Type‐I error accumulation, we employed a cross‐validation approach. However, we also provide in the Supplementary Material (in supporting information) the FDR‐corrected values for each test. Specifically, across the primary cross‐sectional and longitudinal analyses described above (four pPRS x three Braak stage ROIs per model), *p* values were corrected within each analysis set using the Benjamini–Hochberg false discovery rate (FDR) procedure (*p*_FDR < 0.05), chosen over more conservative approaches (e.g., Bonferroni) given the expected correlation between pPRS and between adjacent Braak stage ROIs. In ADNI, our discovery cohort, correction was applied across all four pPRS and three Braak stage ROIs, treating cross‐sectional and longitudinal analyses as separate sets of 12 tests each. In A4, our validation cohort, analyses were restricted a priori to a confirmatory test of the transport pPRS identified in ADNI; correction was accordingly applied only across the three Braak stage ROIs for this pathway, again treating cross‐sectional and longitudinal analyses as comprising three tests each. The sensitivity analyses and sex‐stratified/PS‐matched analyses described above were considered robustness checks and exploratory/hypothesis‐generating analyses, respectively, consistent with their post hoc rationale, and uncorrected *p* values are reported.

All statistical analyses were conducted in R (version 4.4.0, 2024‐04‐24). Statistical significance was defined as two‐sided *p* < 0.05, and standardized regression coefficients (β) are reported to facilitate interpretation across models. Demographic and biomarker characteristics for both cohorts are summarized in Table 1. Detailed matching parameters and full model specifications are provided in the Supplementary Material in supporting information.

**TABLE 1:** Baseline demographic and clinical characteristics of the ADNI and A4 cohorts.

Characteristic	ADNI (*N* = 441)	A4 (*N* = 250)
Age, years	75.1 ± 7.6	72.0 ± 4.7
Female sex, *n* (%)	236 (53.5)	151 (60.4)
Education, years	16.2 ± 2.4	16.2 ± 2.6
*APOE* ε4 carriers, *n* (%)	243 (55.1)	145 (58.0)
CU, *n* (%)	202 (45.8)	250 (100)
MCI, *n* (%)	162 (36.7)	0
Dementia, *n* (%)	77 (17.5)	0
Amyloid PET SUVR (Centiloid)	68.5 ± 36.7	71.1 ± 31.8
Tau positivity (T+), *n* (%)	165 (37.4)	61 (24.4)
AV1451 SUVR, Braak I	1.3 ± 0.3	1.14 ± 0.18
AV1451 SUVR, Braak III–IV	1.2 ± 0.2	1.12 ± 0.08
AV1451 SUVR, Braak V–VI	1.2 ± 0.2	1.10 ± 0.07
Amyloid chronicity, years	12.4 ± 9.3	13.2 ± 7.4
Estimated amyloid age at onset, years	62.7 ± 11.2	58.4 ± 7.5

*Note*: Baseline characteristics of the ADNI (*n* = 441) and A4 cohorts (*n* = 250) are shown. Tau positivity (T+) was defined as meta‐temporal tau SUVR > 1.30. Amyloid chronicity and estimated amyloid age at onset were derived using the SILA algorithm. Chronicity reflects time since estimated amyloid positivity, whereas age at onset represents the estimated age at which amyloid accumulation began. The ADNI cohort included amyloid‐positive participants across the cognitive spectrum (CU, MCI, dementia), whereas the A4 replication cohort consisted of cognitively unimpaired, amyloid‐positive older adults. Values are presented as mean ± standard deviation unless specified otherwise.

Abbreviations: A4, Anti‐Amyloid Treatment in Asymptomatic Alzheimer's Disease; AD, Alzheimer's disease; ADNI, Alzheimer's Disease Neuroimaging Initiative; APOE, apolipoprotein E; CU, cognitively unimpaired; MCI, mild cognitive impairment; PET, positron emission tomography; PRS, polygenic risk score; SILA, sampled iterative local approximation; SUVR, standardized uptake value ratio.

## 3 RESULTS

### 3.1 Genetic risk variants map onto biological pathways

Hierarchical clustering of semantically related biological processes revealed four distinct and biologically coherent pathway clusters (Figure 1). These clusters captured key mechanisms implicated in AD, corresponding to adaptive immune response, leukocyte activation, transport, and intracellular signaling. A detailed list of the top GO BPs contributing to each cluster is provided in Table S2 in supporting information.

**FIGURE 1:**
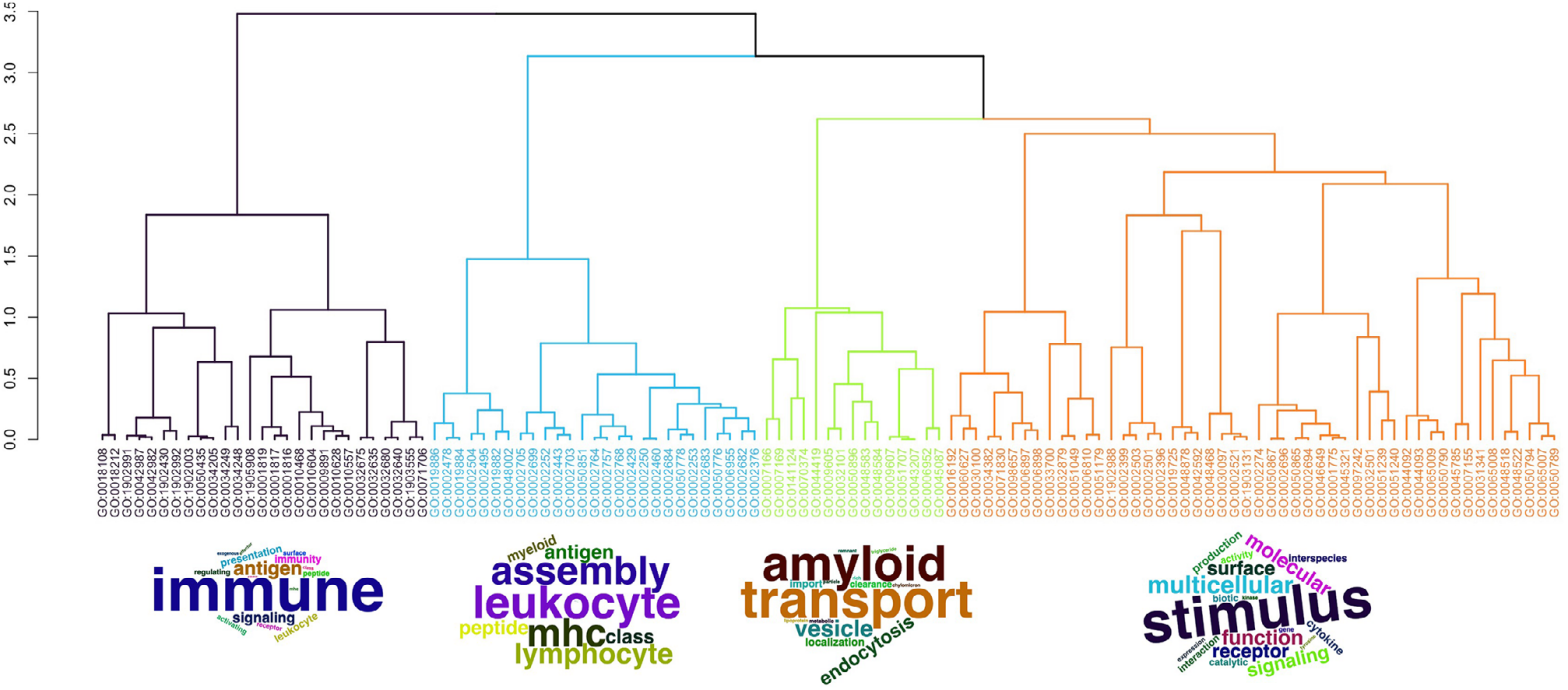
Results from the hierarchical clustering. Gene Ontology (GO) biological pathways were grouped into four biologically relevant clusters based on single nucleotide polymorphism to pathway mapping: adaptive immune response, leukocyte activation, transport, and intracellular signaling. Word clouds visualize the most frequent terms from the pathway descriptions within each cluster.

Overall, 254 SNPs mapped to 212 genes, with contributions distributed across the four clusters: adaptive immune response (*n* = 88), leukocyte activation (*n* = 55), transport (*n* = 105), and intracellular signaling (*n* = 157). These clusters formed the basis for subsequent pPRS computation. The leading genes within each cluster, identified by their relative mapping weight, are illustrated in Figure 2.

**FIGURE 2:**
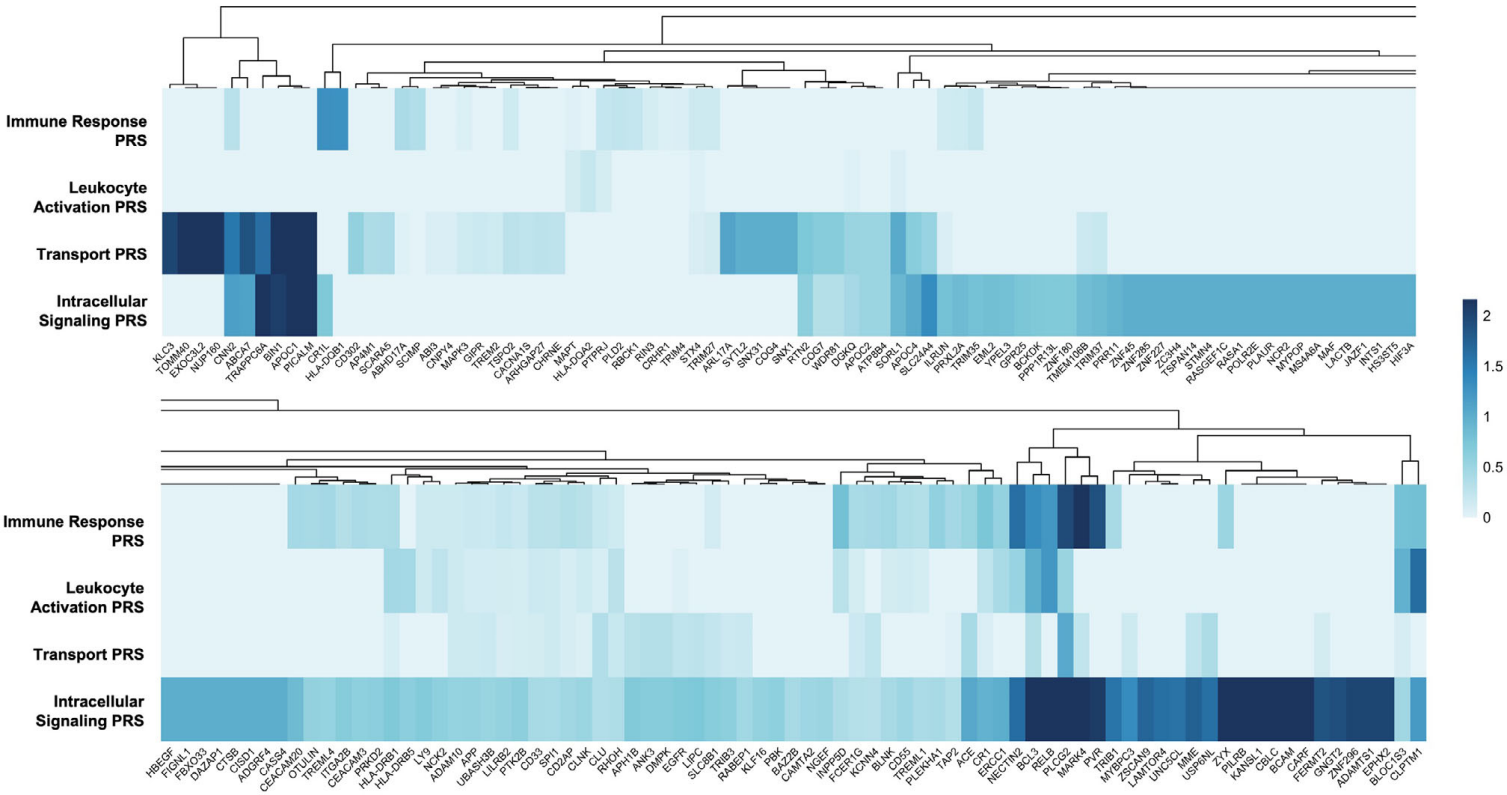
Pathway–gene clustering of AD‐associated variants across four pPRS clusters. Heatmap shows the distribution of AD‐associated SNPs across the four pathway clusters derived from gene‐set enrichment analyses: adaptive immune response PRS, leukocyte activation PRS, transport PRS, and intracellular signaling PRS. Each row corresponds to a gene, and each column represents a pPRS cluster. Colors reflect each gene's pathway mapping weight within a cluster (the summed SNP‐to‐cluster–weighted mapping factor across all SNPs assigned to that gene), with dark blue indicating a stronger contribution to a given pathway and light blue indicating a weaker contribution. Hierarchical clustering highlights shared versus unique genetic contributions across pathways. AD, Alzheimer's disease; pPRS, pathway‐specific polygenic risk score; PRS, polygenic risk score; SNP, single nucleotide polymorphism.

### 3.2 Pathway‐specific polygenic risk modulates amyloid‐driven tau deposition

In ADNI, the interaction between transport pPRS and amyloid chronicity showed a Braak stage–dependent pattern. Specifically, higher transport pPRS scores were associated with a stronger relationship between amyloid chronicity and tau PET deposition in Braak I (β = 0.02, *t* = 2.02, df = 432, *p* = 0.044, Δ*R* ^2^ = 0.006), with results being highly robust in Braak III and IV (β = 0.02, *t* = 2.92, df = 432, *p* = 0.004, Δ*R* ^2^ = 0.011) and V and VI (β = 0.02, *t* = 3.07, df = 432, *p* = 0.002, Δ*R* ^2^ = 0.017; Figure 3, upper row; Table S3 in supporting information). In the A4 cohort, which consisted exclusively of CU amyloid‐positive individuals, an interaction effect of transport pPRS by amyloid chronicity was observed in Braak I (β = 0.018, *t* = 2.00, df = 239, *p* = 0.047, Δ*R* ^2^ = 0.003; Table S3). No interactions with other pathway‐specific risk scores reached significance in both cohorts (*p* > 0.05).

**FIGURE 3:**
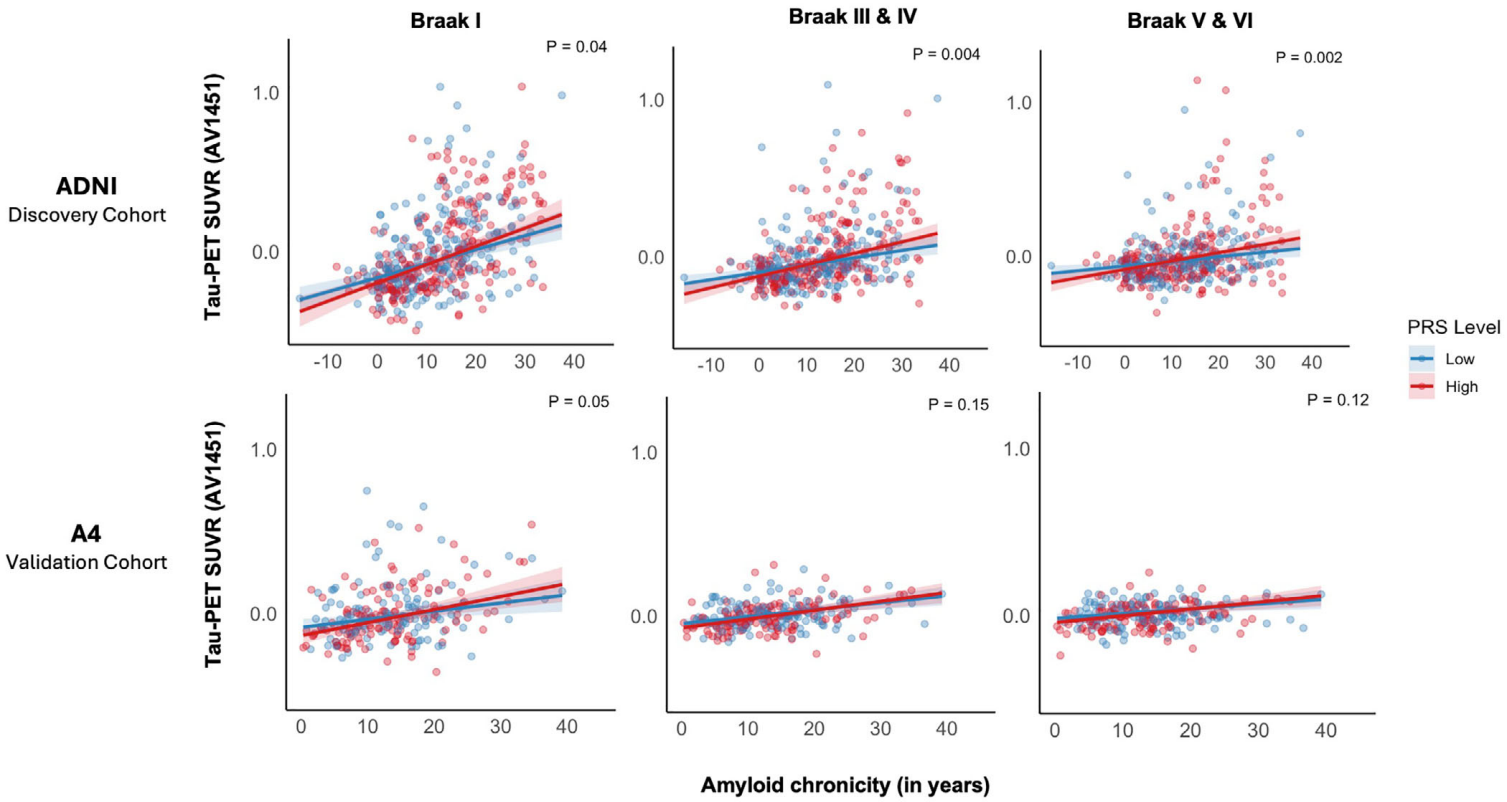
Transport pPRS modulates the association between amyloid chronicity and tau deposition. Residual regression scatterplots show the interaction between amyloid chronicity (*x* axis) and transport pPRS on tau PET SUVR across Braak stage regions (columns) in the ADNI (top row) and A4 cohorts (bottom row). Regression lines and 95% CI (shaded areas) are presented for binarized groups defined by pPRS < 25th percentile (blue, “Low pPRS”) and > 75th percentile (red, “High pPRS”). Note that the binarization of the groups was done for illustration purposes only, and the continuous pPRS values were included in the regression analyses. A4, Anti‐Amyloid Treatment in Asymptomatic Alzheimer's Disease; ADNI, Alzheimer's Disease Neuroimaging Initiative; CI, confidence interval; PET, positron emission tomography; pPRS, pathway‐specific polygenic risk score; SUVR, standardized uptake value ratio.

The transport PRS was driven by genes with high relative mapping weight (Figure 2), predominantly involved in intracellular transport (*KLC3, EXOC3L3, TRAPPC10, NUP160, SORL1, SNX1, SNX31, COG4, SYTL2, ARL17A*), as well as vesicle trafficking (*BIN1, PICALM*) and lipid transport (*ABCA7, APOC1, TOMM40*).^46^, ^47^, ^48^, ^49^, ^50^, ^51^, ^52^, ^53^ These findings suggest that individuals with elevated genetic risk in transport pathways exhibit greater amyloid chronicity–dependent tau accumulation in cortical regions.

To confirm robustness of our findings with respect to the influence of potentially influential observations, we repeated our regression analyses after excluding participants whose Cook distance exceeded the threshold of 4/*n* within each Braak region and PRS model (59 excluded, *n* = 382 remaining, in ADNI, in which influential observations were pooled across all four pPRS; 26 excluded, *n* = 224 remaining, in A4, in which detection was restricted to the transport pPRS given its role as the sole confirmatory pathway tested in that cohort). The result pattern remained unchanged: in ADNI, interaction effects between the transport pPRS and amyloid chronicity persisted in Braak III and IV (β = 0.0114, *t* = 2.05, df = 373, *p* = 0.042) and V and VI (β = 0.0143, *t* = 2.94, df = 373, *p* = 0.003), with no significant effect at Braak I (β = 0.0107, *t* = 1.28, df = 373, *p* = 0.200), while in A4, the same pathway showed a consistent effect limited to Braak I (β = 0.0254, *t* = 2.16, df = 213, *p* = 0.032), with no significant effects at Braak III and IV or V and VI (Figure S3 in supporting information).

### 3.3 Pathway‐specific genetic modulation of longitudinal tau accumulation

Next, we tested whether amyloid chronicity and pathway‐specific genetic risk were associated with longitudinal changes in tau PET deposition. In ADNI (*n* = 224), at higher levels of transport pPRS there was a stronger association between baseline amyloid chronicity and longitudinal tau PET increase in Braak III and IV (β = 0.0033, *t* = 2.68, df = 215, *p* = 0.008, Δ*R* ^2^ = 0.020) and Braak V and VI (β = 0.0032, *t* = 3.09, df = 215, *p* = 0.002, Δ*R* ^2^ = 0.023, Figure 4; Table S3), but only at trend level in Braak I (β = 0.0016, *t* = 1.89, df = 215, *p* = 0.060, Δ*R* ^2^ = 0.011, Figure 4).

**FIGURE 4:**
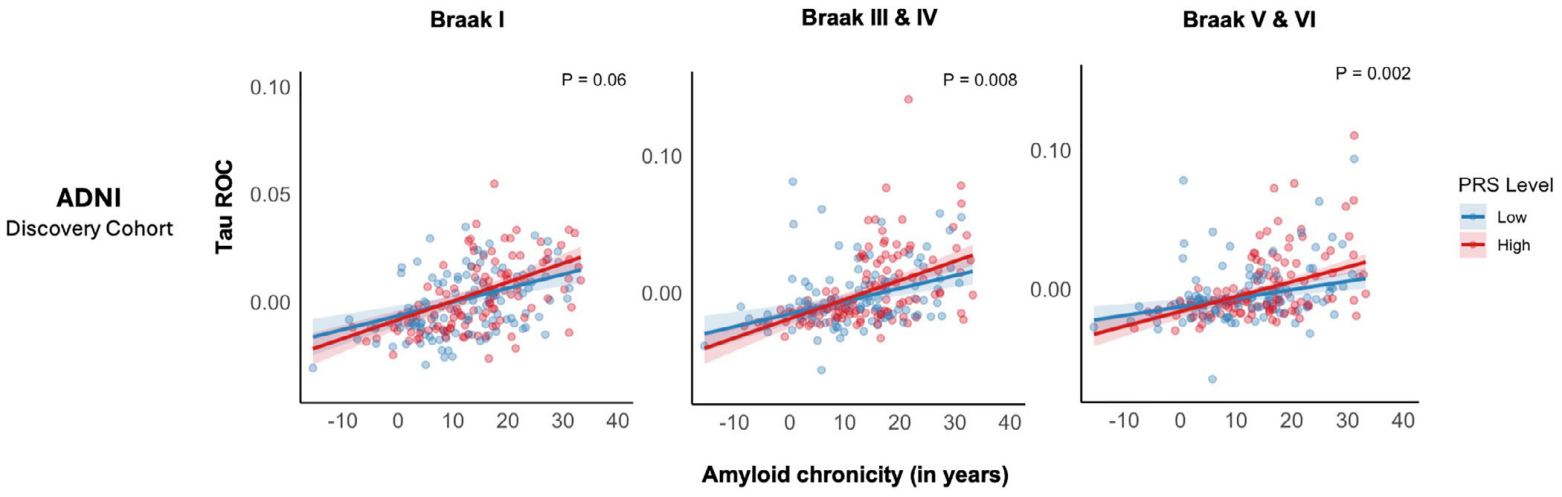
pPRSs moderate the longitudinal association between amyloid chronicity and tau ROC. Residual regression scatterplots show the interaction between baseline amyloid chronicity (*x* axis) and transport pPRS on longitudinal tau PET SUVR ROC across Braak stage regions (columns). Regression lines represent participants at the 25th percentile (blue, “Low pPRS”) and 75th percentile (red, “High pPRS”) of pathway‐specific genetic risk. Significant interaction effects were observed in the ADNI cohort (first row) on Braak regions III and IV and V and VI (*p* < 0.01), with a trend‐level effect in Braak I (*p* = 0.05). ADNI, Alzheimer's Disease Neuroimaging Initiative; PET, positron emission tomography; pPRS, pathway‐specific polygenic risk score; ROC, rate of change; SUVR, standardized uptake value ratio.

Sensitivity analyses excluding influential observations based on Cook distance yielded consistent estimates for Braak III and IV (β = 0.0036, *t* = 3.05, df = 184, *p* = 0.003) and Braak V and VI (β = 0.0029, *t* = 2.97, df = 184, *p* = 0.003; Figure S4 in supporting information).

We next examined longitudinal tau accumulation in the A4 subset with follow‐up tau PET (*n* = 113). In line with the broader pattern observed across both ADNI cross‐sectional and longitudinal analyses, as well as the A4 cross‐sectional findings, the transport pPRS showed a positive interaction with amyloid chronicity across Braak stage regions; however, this association did not reach statistical significance in this preclinical A4 longitudinal subset (Table S3). Notably, the effect sizes were comparable to, or slightly larger than, those observed cross‐sectionally, suggesting that the smaller longitudinal sample and the minimal tau progression, characteristic of A4, likely limited statistical power to detect these associations (Figure S5 in supporting information).

### 3.4 Sex‐specific genetic effects

Given evidence for sex differences in AD pathophysiology,^17^, ^18^, ^19^ we next examined whether pathway‐specific genetic modulation of amyloid‐related tau PET uptake differed between females and males (Table S4 in supporting information).

In sex‐stratified analyses in PS‐matched female and male groups of participants with AD in ADNI (*n* = 169 females; *n* = 169 males), the interaction effect of transport pPRS by amyloid chronicity on tau PET was significant for females in Braak I (β = 0.020, *t* = 2.32, df = 162, *p* = 0.022; Figure S6 in supporting information) and Braak III and IV (β = 0.021, *t* = 3.44, df = 162, *p* < 0.001; Figure S6), with a trend in Braak V and VI (β = 0.020, *t* = 1.83, df = 162, *p* = 0.069; Figure S6). In males, this association was significant only in Braak III and IV (β = 0.032, *t* = 2.03, df = 162, *p* = 0.044; Figure S6). In males, no other interactions were significant for any pathway‐specific PRS at any Braak region (all *p* > 0.05; Table S5 in supporting information).

The same sex‐stratified analysis in the A4 cohort (*n* = 86 females and *n* = 86 males, propensity matched) showed a significant interaction effect for transport pPRS on tau PET deposition in Braak I (β = 0.026, *t* = 2.73, df = 79, *p* = 0.008; Figure S6), in females but not males (β = 0.004, *t* = 0.12, df = 79, *p* = 0.906; Figure S6). This effect was not observed at higher Braak stage ROIs in females (Braak III–IV: β = 0.012, *t* = 1.61, df = 79, *p* = 0.111; Braak V–VI: β = 0.011, *t* = 1.52, df = 79, *p* = 0.132 Table S5), and no interactions were significant in males at any Braak region for this pathway (Table S5). This significant region in A4 (Braak I) corresponds to one of the two regions where transport pPRS was significant in females in ADNI, providing partial convergence between the two cohorts, though the ADNI female effect additionally extended to Braak III and IV, which was not observed in A4.

### 3.5 Sensitivity and exploratory analyses of the transport pPRS effects

To assess whether the transport pPRS x amyloid chronicity interaction reflects genuine effect modification rather than two independent main effects, we examined whether the transport pPRS shows a direct association with amyloid burden and with tau PET deposition, and additionally tested amyloid chronicity for a direct association with tau PET deposition, independent of pPRS (Tables S6–S8 in supporting information). The transport pPRS was associated with neither SILA‐derived amyloid chronicity nor Centiloid values in either ADNI or A4 (Table S6), nor with tau PET deposition at any Braak stage ROI in ADNI or at Braak I or V and VI in A4, except for a nominal association at Braak III and IV in A4 (Table S7). In contrast, amyloid chronicity itself was robustly associated with tau PET burden at all Braak stage ROIs in the ADNI and A4 cross‐sectional analyses (all *p* < 0.001); longitudinally these effects persisted for ADNI and were significant in A4 in Braak III and IV (*p* = 0.002) and V and VI (*p* = 0.020), though not Braak I (Table S8).

To determine whether the observed associations depended on the use of SILA‐derived amyloid chronicity, we repeated the primary analyses using amyloid PET Centiloid values as an alternative measure of amyloid burden. In ADNI, the transport pPRS x Centiloid interaction showed a pattern consistent with the primary SILA‐based analysis, with significant cross‐sectional effects in Braak III and IV (β = 0.0044, *t* = 2.89, *p* = 0.004) and Braak V and VI (β = 0.0040, *t* = 3.06, *p* = 0.002), and a trend‐level effect in Braak I (β = 0.0042, *t* = 1.86, *p* = 0.064; Table S9 in supporting information). In A4, the Centiloid‐based analysis similarly showed a significant interaction in Braak I (β = 0.0045, *t* = 2.15, *p* = 0.033), but not in the higher Braak stage ROIs (Table S9). Longitudinal analyses using Centiloid values in ADNI likewise yielded significant transport pPRS x Centiloid interactions in Braak III and IV (β = 0.00097, *t* = 2.95, *p* = 0.0036) and Braak V and VI (β = 0.00107, *t* = 3.83, *p* < 0.001), whereas no significant effects were observed in the A4 longitudinal sample (all *p* ≥.070; Table S9). Thus, the longitudinal Centiloid analyses reproduced the regional pattern observed in the primary SILA‐based models in ADNI, indicating that the transport pPRS association was robust to the choice of amyloid measure.

We next assessed the effects of the transport pPRS when informed by the *APOE* ε4 genotype. The transport pPRS was recalculated by adding the weights of the *APOE* locus variants, and the regression analyses on the pPRS x amyloid chronicity interaction were repeated. In ADNI, the transport pPRS (*APOE* included) x amyloid chronicity association remained significant in all Braak stage ROIs (all *p* < 0.001; Table S10 in supporting information). In A4, the same interaction was significant at the higher composite Braak stage ROIs (III–IV: *p* = 0.035; V–VI: *p* = 0.027; Table S10) but not at Braak I. No A4 longitudinal associations reached significance for the transport pPRS, and the remaining three pathway scores were non‐significant across all A4 analyses.

There was no evidence of a hemisphere‐dependent effect of pPRS, as the pPRS x amyloid chronicity x hemisphere interaction was not significant in all pathways. We additionally tested whether pPRS scores differed across data‐driven tau PET spatial subtypes.^43^, ^44^ None of the four pathway‐specific pPRS scores differed significantly between tau PET spatial subtypes, with consistent null findings across different subtype‐assignment probability thresholds (Figure S7 in supporting information). Together, these findings suggest that the observed pathway‐specific genetic effects were not associated with systematic differences in the hemispheric or broader spatial distribution of tau PET pathology, but rather reflected the transport pPRS x amyloid chronicity effect tracking the normative Braak‐staged topography of AD pathology rather than a spatially restricted process.

## 4 DISCUSSION

Our major finding was that individuals with higher transport pPRSs exhibited stronger baseline associations between amyloid chronicity and regional tau PET uptake, with more pronounced effects in females than males. Longitudinally, a higher transport pPRS was associated with a greater rate of tau PET accumulation in participants with symptomatic AD. Collectively, these findings suggest that genetic variation in transport pathways amplifies amyloid‐driven tau deposition.

Our findings support previous reports suggesting that polygenic risk acts in a biological pathway–specific manner on AD pathologies.^5^, ^8^, ^11^, ^13^, ^14^, ^34^, ^54^ We derived our four pathway clusters empirically via semantic clustering of enriched GO BP terms, allowing pathway structure to emerge directly from the GWAS‐implicated gene set rather than from a predefined pathway taxonomy. In contrast to some previous studies,^8^, ^11^, ^13^ we did not identify a cluster defined by amyloid deposition or clearance. However, amyloid‐related terms were not absent from our semantic clustering: the transport pPRS cluster included not only broad terms such as transport, vesicle‐mediated transport, endocytosis, and regulation of transport, but also terms linked to Aβ metabolic process and amyloid precursor protein (APP) catabolic process. Thus, amyloid‐related processes were captured within a broader intracellular transport and endocytic signature rather than forming an independent amyloid‐specific cluster.

The significant transport pPRS effect at Braak I in a second, independently recruited, preclinical cohort, suggests that these genetic influences may be detectable even at this early stage. That the effect was restricted to Braak I and did not extend to higher cortical regions in A4 likely reflects this preclinical sample's limited cortical tau spread (Figure S5), whereas ADNI showed the same Braak I effect cross‐sectionally, alongside a more robust transport pPRS x amyloid‐chronicity interaction at higher Braak stages (III–IV, V–VI). Longitudinal trends in A4 were directionally consistent with ADNI but did not reach significance, potentially reflecting limited power given this sample's minimal tau progression.

In sensitivity analyses, we repeated our analyses replacing amyloid chronicity by Centiloids, obtaining largely consistent effects of the pPRS on tau PET. These findings suggest that the result patterns do not depend on the SILA estimated chronicity. In principle, Centiloid and SILA capture related but distinct aspects of the accumulation of amyloid pathology: amyloid chronicity relates tau PET accumulation to the duration of amyloid positivity,^15^ which is useful once amyloid accumulation plateaus, because chronicity still increases with disease duration even as amyloid PET accumulation levels off.^55^ Our finding of consistent pPRS interaction effect regardless of measure likely reflects that most subjects fell in a time period during which amyloid PET still increases significantly, so that amyloid chronicity and Centiloid may have been highly correlated and conveyed similar information.

Our transport pPRS genes overlap with those included in a previously reported “clearance” pPRS in patients with AD,^8^ which was strongly weighted by genes such as *SNX1* and *BIN1*. Alterations in protein sorting and endosomal recycling have been associated with altered APP processing and thus may enhance amyloidogenic pathways.^46^, ^47^, ^48^, ^50^, ^51^, ^52^, ^56^ At the same time, vesicular trafficking and endocytosis such as regulated by *BIN1* may also play a key role in transmitting tau between cells, and an AD risk SNP in *BIN1* has been previously associated with increased tau PET deposition in AD.^57^, ^58^, ^59^ Together these findings support a role for intracellular trafficking and vesicular transport in key AD pathologies.

Other SNPs ranking high in the transport pPRS were located in the *ABCA7* and *APOC1* genes, which are involved in lipid‐mediated amyloid clearance and lipoprotein exchange,^53^, ^60^ and in the *TOMM40* gene, which is involved in mitochondrial trafficking that can contribute to both Aβ accumulation and tau phosphorylation if impaired.^49^, ^54^, ^60^ Although the *APOC1* and *TOMM40* genes are both in the chromosome 19 neighborhood of *APOE*,^61^, ^62^ and *TOMM40* shows moderately high LD with *APOE*,^61^ our effects are unlikely to be solely attributable to the *APOE* genotype. This is supported by LD‐based clumping (*R* ^2^ < 0.1, 250 kb, 1000G EUR reference) during score construction to reduce correlated‐variant signal, exclusion of variants annotated directly to *APOE*, and inclusion of the *APOE* ε4 genotype as a covariate in our regression analyses. Furthermore, adding *APOE* ε4 genotype to the pPRS calculation did not alter the result pattern. While the locus’ complex LD structure and physical connections between these genes could influence regional gene expression nearby,^61^, ^62^ our findings support a role for lipid‐processing and mitochondrial pathways, not exclusively modulated by *APOE* ε4, in tau pathology development.^13^, ^49^, ^50^, ^53^, ^54^, ^56^ Consistent with this, the transport pPRS x amyloid‐chronicity association was already significant at all Braak stage ROIs in ADNI, and adding *APOE* locus variants to the score strengthened rather than newly produced this effect (Table S10).

Motivated by previous findings of increased tau PET deposition in females compared to males,^17^, ^18^, ^19^ we explored whether polygenic risk is associated with higher tau PET accumulation. Despite the relatively small sex‐stratified sample sizes, transport pPRS associations with tau PET deposition were more pronounced in females than males, generating the hypothesis that sex difference in APP trafficking and endosomal–vesicular transport contribute to tau pathology, and thus may heighten vulnerability in women despite comparable amyloid exposure.^17^, ^18^, ^19^

While our observations reflect system‐level genetic effects, these prior molecular findings on APP‐directed endosomal, Golgi, and mitochondrial trafficking networks provide a potential explanation for the observed association between transport pPRS and amyloid‐related development of pathological tau in AD.

## 5 LIMITATIONS

Several limitations should be noted. Differences in genotyping coverage and imputation quality between ADNI and A4 reduced SNP overlap, limiting replication across samples. Because each pathway‐specific PRS is a weighted sum across mapped SNPs, this differential attrition could alter a pathway score's relative genetic composition between cohorts independent of true biological differences, and may have contributed to the more modest, spatially restricted transport pPRS effects in A4, alongside its smaller sample size. Effect sizes were also relatively small given typical PRS effects on AD biomarkers; transport pPRS explained up to 1.7% of tau PET variance in discovery and ≈ 0.4% in validation, within the range of previous pPRS studies, but this modest effect size may explain the failure to replicate associations for other pathways such as immunity,^8^, ^11^, ^13^ especially when stratifying by sex. A further limitation concerns the granularity of our pathway definitions. Broader clusters such as transport pPRS aggregate genes spanning mechanistically distinct sub‐processes (e.g., APP processing alongside general vesicular trafficking), limiting our ability to isolate which sub‐processes drive the observed tau association. Resolving pathway clusters into narrower, adequately powered subcomponents is a direction for future work in larger cohorts.

The predominantly White/European, highly educated composition of both samples may further limit generalizability, and PRSs’ ancestry‐related sensitivity highlights the need for replication in multi‐ethnic samples.^63^ Our polygenic scores were also derived only from a meta‐analysis of Wightman et al.^3^ and Bellenguez et al.^4^ without additional GWAS sources such as Schwartzentruber et al.;^64^ incorporating further summary statistics, with appropriate accounting for sample overlap, is a direction for future refinement.

SILA‐derived amyloid chronicity assumes a single population‐level amyloid trajectory shared across participants, which genetic risk affecting amyloid dynamics could in principle bias; nonetheless, transport pPRS showed no significant direct association with amyloid burden in either cohort or amyloid measure, and our findings were consistent using Centiloid, a cohort‐independent measure requiring no trajectory modeling, in place of amyloid chronicity (Tables S6 and S9), arguing against both the single‐trajectory assumption and differential cohort comparability as drivers of the more modest A4 effects. A4's exclusively CU composition and comparable‐to‐higher amyloid PET burden relative to ADNI further raises the possibility that cognitive resilience or unmeasured factors such as vascular burden and co‐pathology differ between cohorts and differentially influence the amyloid–tau relationship, a residual contribution of such unmeasured factors cannot be fully excluded,^65^ and future work incorporating vascular imaging and co‐pathology biomarkers would help clarify this. Furthermore, previous studies reported pPRS scores including AD risk variants to be associated with volumetric gray matter changes even in young and older non‐demented individuals.^66^ Future studies may explore how early pre‐morbid brain changes driven by AD risk variants may predispose to AD risk.

## 6 CONCLUSIONS

We provide genetic evidence, most robust in a well‐powered discovery cohort and directionally consistent in an independent cohort, implicating intracellular transport pathways in tau accumulation in AD. By integrating subject‐specific estimates of tau accumulation with a clock model of amyloid progression, we were able to reveal a genetically modulated, time‐dependent coupling between these two AD core pathologies. While pPRSs do not lend themselves to risk stratification in personalized medicine due to the limited effect size, they are nevertheless instrumental for the discovery of potential biological pathways that drive tau pathology and that could be targeted by therapeutic intervention.

## AUTHOR CONTRIBUTIONS

**Carolina Valentim** and **Michael Ewers**: study concept and design, data analysis, and drafting of the manuscript. **Simon Frerich**, **Rainer Malik**, **Jannis Denecke**, and **Nicolai Franzmeier**: data processing and critical revision of the manuscript. **Yuanxi Li**, **Reihaneh Ahmadi** and **Tobey J. Betthauser**: critical revision of the manuscript.

## CONFLICT OF INTEREST STATEMENT

The authors declare no conflicts of interest. Author disclosures are available in the Supporting Information.

## CONSENT STATEMENT

All human subjects provided informed consent.

## Supporting information




Supporting Information

Supporting Information

## Data Availability

All data associated with this study are present in the paper or the Supplementary Materials. ADNI and A4 data are publicly available after registration on the study websites (ADNI: https://adni.loni.usc.edu/about/adni4/; A4: www.a4studydata.org).
