# Supplementary material for: Pathway‐specific polygenic risk scores modify the association between amyloid and tau PET accumulation in patients with Alzheimer's disease
Source: Alzheimers Dement. 2026 Oct 27;22(10):e71939. doi: 10.1002/alz.71939 (PMC13711249; doi:10.1002/alz.71939)
Supplement: Supplementary file 2 [file ALZ-22-e71939-s002.docx]

**SUPPLEMENTARY MATERIAL**

A detailed pathway selection criteria, enrichment statistics, clustering procedures, PRS construction code are available in the public GitHub repository: <https://github.com/c-valenti/PathwayPRS_AD> .

**Supplementary Figures**

**Figure S1.** **Participant selection flow diagram for ADNI and A4 cohorts.** Flowchart illustrates inclusion criteria and stepwise sample selection for the discovery cohort (ADNI) and validation cohort (A4). From the initial ADNI sample (n = 3674), participants were included if they had at least one tau-PET scan acquired within 30 days of an amyloid-PET scan. The final ADNI discovery cohort consisted of 441 amyloid-positive participants with baseline tau-PET and genetic data, and 224 with longitudinal tau-PET data. In A4, from 591 placebo participants, 250 cognitively normal, amyloid-positive individuals with available tau-PET and genetic data were retained as the validation cohort and 113 for longitudinal analyses.

**Figure S2. SILA model fits for amyloid accumulation in ADNI cohort.** Scatterplot shows centiloid values as a function of amyloid chronicity (years since estimated amyloid positivity threshold of centiloid > 21), with each line representing an individual participant (n = 3,674; 15,250 sessions). Among participants with available amyloid PET data (n = 1,642), 904 were amyloid-positive (A+: centiloid > 21) and 738 were amyloid-negative (A-) at their earliest PET visit. The population-level trajectory curve (bold line) captures the characteristic sigmoidal pattern of amyloid accumulation over time. Negative values on the x-axis indicate estimated years until amyloid positivity is predicted to be reached.

**Figure S3. Associations between amyloid chronicity and tau-PET remain robust after exclusion of influential observations.** Residual regression scatterplots illustrate the interaction between amyloid chronicity (x-axis) and transport pathway-specific polygenic risk scores (pPRS) on regional tau-PET SUVR (AV1451) across Braak-stage ROIs in the ADNI discovery cohort (top row) and the A4 validation cohort (bottom row), stratified by high vs. low transport pPRS. Regression lines with 95% confidence intervals are shown for illustration using binarized pPRS groups (<25th percentile = blue, "low pPRS"; >75th percentile = red, "high pPRS"), although all analyses were performed using continuous pPRS values. Results were consistent with the main analyses, confirming that observed associations between amyloid chronicity, genetic risk, and tau accumulation were not driven by individually influential observations.

**Figure S4. Influential observation exclusion confirms longitudinal modulation of amyloid-tau associations by transport pPRS.** Residual regression scatterplots illustrate the interaction between amyloid chronicity (x-axis) and transport pathway-specific polygenic risk scores (pPRS) on longitudinal rate of change in tau-PET (AV1451) SUVR across Braak-stage regions (columns) in the ADNI discovery cohort. Regression lines with 95% confidence intervals are shown for illustration using binarized pPRS groups (<25th percentile = blue, "low pPRS"; >75th percentile = red, "high pPRS"), although all analyses were performed using continuous pPRS values. Results remained consistent with the main analyses, confirming that observed associations between amyloid chronicity, genetic risk, and longitudinal tau accumulation were not driven by individually influential observations.

**Figure S5.** **Global distribution of tau-PET SUVR in discovery and validation cohorts.** Surface maps display average tau-PET standardized uptake value ratios (SUVRs) across cortical regions in the ADNI discovery cohort (top row) and the A4 validation cohort (bottom row). Darker colors indicate higher tau burden. Compared with ADNI, A4 participants,who were exclusively cognitively unimpaired but amyloid-positive, showed globally lower tau uptake, with early involvement primarily in medial temporal and adjacent regions.

**Figure S6. Sex-specific propensity-score matched analyses in ADNI and A4.** Residual regression scatterplots show the interaction between amyloid chronicity and transport pPRS on tau-PET SUVR across Braak-stage regions, displayed separately for females (top panels) and males (lower panels). Regression lines and 95% CI are shown for illustration purposes.

**A	B**

**Figure S7. SuStaIn K=4 tau-PET spatial subtypes in Aβ-positive ADNI participants: regional tau burden and transport pPRS association, at assignment-confidence ≥0.75. (A)** Regional tau-PET burden by spatial subtype. Group-averaged, regional z-scored tau-PET SUVR (mean, DK atlas parcellation) for each of the four SuStaIn-derived spatial subtypes, restricted to subjects with subtype assignment probability ≥0.75 (Subtype 1, n=33; Subtype 2, n=51; Subtype 3, n=34; Subtype 4, n=21). Warmer colors indicate higher regional tau burden (z-score scale, 0–5). Subtypes are numbered arbitrarily by the SuStaIn algorithm and do not reflect severity or disease stage ordering; **(B)** Transport pPRS by spatial subtype. Boxes show median and interquartile range; points show individual subjects. Transport pPRS did not differ significantly across subtypes, all p≥.318 (covariate-adjusted ANCOVA).

**Supplementary Tables**

**Table S1.** Interaction effects of Global pPRS x amyloid chronicity on tau-PET Braak-stage deposition, ADNI and A4 (comparative).

| **Cohort** | **Timepoint** | **Outcome** | **β** | **SE** | **df** | **t** | **p** | **R²** | **ΔR²** |
| --- | --- | --- | --- | --- | --- | --- | --- | --- | --- |
| **ADNI** | Cross-sectional (N=441) | Braak I | 0.0062 | 0.0089 | 432 | 0.70 | .484 | 0.320 | 0.001 |
|  |  | Braak III-IV | 0.0109 | 0.0062 | 432 | 1.76 | .079 | 0.203 | 0.006 |
|  |  | Braak V-VI | 0.0103 | 0.0053 | 432 | 1.94 | .053 | 0.157 | 0.008 |
|  | Longitudinal (N=224) | Braak I | 0.0017 | 0.0008 | 215 | 2.03 | **.044** | 0.329 | 0.011 |
|  |  | Braak III-IV | 0.0034 | 0.0012 | 215 | 2.87 | **.005** | 0.281 | 0.018 |
|  |  | Braak V-VI | 0.0029 | 0.0010 | 215 | 2.94 | **.004** | 0.228 | 0.014 |
| **A4** | Cross-sectional (N=250) | Braak I | -0.0043 | 0.0093 | 239 | -0.46 | .649 | 0.100 | 0.001 |
|  |  | Braak III-IV | -0.0012 | 0.0049 | 239 | -0.24 | .813 | 0.103 | 0.0004 |
|  |  | Braak V-VI | 0.0016 | 0.0046 | 239 | 0.34 | .734 | 0.077 | 0.0001 |
|  | Longitudinal (N=113) | Braak I | -0.0007 | 0.0011 | 93 | -0.69 | .491 | 0.064 | 0.004 |
|  |  | Braak III-IV | -0.0010 | 0.0013 | 93 | -0.73 | .465 | 0.148 | 0.013 |
|  |  | Braak V-VI | -0.0006 | 0.0011 | 93 | -0.57 | .571 | 0.096 | 0.010 |

*Note.* β = unstandardized Global PRS x amyloid chronicity interaction coefficient from Huber-weighted robust regression, fit on PC-residualized tau-PET Braak-stage values and adjusted for amyloid chronicity, APOE-ε4 x amyloid chronicity interaction, age, sex, and education. N reflects the full cohort; analytic N in individual models may be marginally lower due to listwise deletion of missing covariate data (see df). p-values are reported uncorrected. R² = full-model R²; ΔR² = change in model R² associated with inclusion of the amyloid chronicity x Global pPRS interaction (Huber regression uses data-dependent weights, small negative ΔR² values may occur and indicate no incremental improvement in robust model fit).

**Table S2.** Representative Gene Ontology (GO: BP) biological pathways contributing to each genetic pathway cluster.

| **Cluster** | **GO term name** |
| --- | --- |
| Adaptive Immune PRS | immune system process |
|  | immune response |
|  | regulation of immune system process |
|  | positive regulation of immune system process |
|  | regulation of immune response |
|  | positive regulation of immune response |
|  | immune effector process |
|  | activation of immune response |
|  | leukocyte mediated immunity |
|  | immune response-regulating signaling pathway |
| Leucocyte Activation PRS | cell activation |
|  | leukocyte activation |
|  | lymphocyte activation |
|  | regulation of cell activation |
|  | positive regulation of cell activation |
|  | myeloid leukocyte activation |
|  | MHC class II protein complex assembly |
|  | MHC protein complex assembly |
|  | peptide antigen assembly with MHC class II protein complex |
|  | peptide antigen assembly with MHC protein complex |
| Transport PRS | transport |
|  | vesicle-mediated transport |
|  | regulation of localization |
|  | import into cell |
|  | regulation of transport |
|  | endocytosis |
|  | regulation of vesicle-mediated transport |
|  | regulation of endocytosis |
|  | amyloid-beta metabolic process |
|  | amyloid precursor protein catabolic process |
| Intracellular Signaling PRS | biological regulation |
|  | regulation of biological process |
|  | regulation of cellular process |
|  | positive regulation of biological process |
|  | positive regulation of cellular process |
|  | regulation of response to stimulus |
|  | regulation of multicellular organismal process |
|  | cell surface receptor signaling pathway |
|  | response to external stimulus |
|  | regulation of molecular function |

*Note.* Top ten highest-weighted Gene Ontology biological process terms contributing to each pathway cluster.

**Table S3.** Interaction effects of Transport pPRS x amyloid chronicity on tau-PET Braak-stage deposition, ADNI and A4.

| **Cohort** | **Timepoint** | **Outcome** | **β** | **SE** | **df** | **t** | **p** | **p_FDR_BH** | **R²** | Δ**R²** |
| --- | --- | --- | --- | --- | --- | --- | --- | --- | --- | --- |
| **ADNI** | Cross-sectional (N=441) | Braak I | 0.0178 | 0.0088 | 432 | 2.02 | **.044** | .175 | 0.322 | 0.006 |
|  |  | Braak III-IV | 0.0177 | 0.0060 | 432 | 2.92 | **.004** | **.022** | 0.206 | 0.011 |
|  |  | Braak V-VI | 0.0161 | 0.0052 | 432 | 3.07 | **.002** | **.022** | 0.164 | 0.017 |
|  | Longitudinal (N=224) | Braak I | 0.0016 | 0.0009 | 215 | 1.89 | .060 | .182 | 0.315 | 0.011 |
|  |  | Braak III-IV | 0.0033 | 0.0012 | 215 | 2.68 | **.008** | **.048** | 0.279 | 0.020 |
|  |  | Braak V-VI | 0.0032 | 0.0010 | 215 | 3.09 | **.002** | **.027** | 0.237 | 0.023 |
| **A4** | Cross-sectional (N=250) | Braak I | 0.0177 | 0.0088 | 239 | 2.00 | **.047** | .140 | 0.107 | 0.003 |
|  |  | Braak III-IV | 0.0067 | 0.0047 | 239 | 1.44 | .151 | .151 | 0.112 | 0.003 |
|  |  | Braak V-VI | 0.0068 | 0.0044 | 239 | 1.54 | .125 | .151 | 0.085 | 0.005 |
|  | Longitudinal (N=113) | Braak I | 0.0016 | 0.0011 | 93 | 1.43 | .155 | .450 | 0.084 | 0.012 |
|  |  | Braak III-IV | 0.0014 | 0.0014 | 93 | 1.04 | .300 | .450 | 0.137 | -0.004 |
|  |  | Braak V-VI | 0.0009 | 0.0011 | 93 | 0.76 | .452 | .452 | 0.091 | -0.004 |

*Note.* β = unstandardized Transport PRS x amyloid chronicity interaction coefficient from Huber-weighted robust regression, fit on PC-residualized tau-PET Braak-stage values and adjusted for amyloid chronicity, APOE-ε4 x amyloid chronicity interaction, age, sex, and education. N reflects the full cohort; N in individual models may be marginally lower due to listwise deletion of missing covariate data (see df). p_FDR_BH = Benjamini-Hochberg-corrected p-values, applied separately within each cohort x timepoint set: ADNI comprises 12 tests each (four pathway-specific PRS x three Braak-stage ROIs); A4 comprises 3 tests each (transport pPRS only x three Braak-stage ROIs). Bold = p < .05. R² = full-model R²; ΔR² = change in model R² associated with inclusion of the amyloid chronicity x pPRS interaction (Huber regression uses data-dependent weights, small negative ΔR² values may occur and indicate no incremental improvement in robust model fit).

**Table S4.** Propensity-matched baseline characteristics of ADNI and A4 participants in the sex-matched analysis.

|  | **ADNI (N = 338)** | **A4 (N = 172)** |
| --- | --- | --- |
| **Female subgroup (N)** | **169** | **86** |
| Age (years) | 74.32 ± 7.78 | 72.35 ± 4.8 |
| Years of education | 16.15 ± 2.26 | 16.19 ± 2.3 |
| Diagnosis CU | 84 (49.7%) | 86 (100%) |
| MCI | 57 (33.7%) | - |
| Dementia | 28 (16.6%) | - |
| APOE-ε4 carriers | 94 (55.6%) | 49 (57%) |
| Amyloid-PET SUVR (Centiloid) | 63.70 ± 35.8 | 69.94 ± 36.7 |
| Tau+ status | 65 (38.5%) | 17 (19.8%) |
| **Male subgroup (N)** | **169** | **86** |
| Age (years) | 75.30 ± 6.93 | 72.53 ± 4.6 |
| Years of education | 16.14 ± 2.42 | 16.45 ± 2.6 |
| Diagnosis CU | 74 (43.8%) | 86 (100%) |
| MCI | 66 (39.1%) | - |
| Dementia | 29 (17.2%) | - |
| APOE-ε4 carriers | 92 (54.4%) | 45 (52.3%) |
| Amyloid-PET SUVR (Centiloid) | 69.75 ± 37.5 | 68 ± 31 |
| Tau+ status | 61 (36.1%) | 24 (27.9%) |

*Note.* Baseline characteristics of the propensity-matched ADNI (n = 338) and A4 (n = 172) subgroups are shown, stratified by sex. The ADNI cohort included amyloid-positive participants across the cognitive spectrum (cognitively unimpaired, mild cognitive impairment, and dementia), whereas the A4 validation cohort consisted of cognitively normal, amyloid-positive older adults. Tau positivity (T+) was defined as meta-temporal tau SUVR > 1.30. Values are presented as mean ± standard deviation unless specified otherwise.

**Table S5.** Sex-stratified propensity-matched interaction effects: cross-sectional (Transport pPRS)

| **Cohort** | **Sex** | **Braak region** | **β** | **SE** | **df** | **t** | **p** |
| --- | --- | --- | --- | --- | --- | --- | --- |
| ADNI (N=338; 169F/169M) | Female | I | 0.0203 | 0.00873 | 162 | 2.32 | **.022** |
|  |  | III-IV | 0.0208 | 0.00605 | 162 | 3.44 | **<.001** |
|  |  | V-VI | 0.0202 | 0.0110 | 162 | 1.83 | .069 |
|  | Male | I | 0.0181 | 0.0151 | 162 | 1.20 | .234 |
|  |  | III-IV | 0.0317 | 0.0156 | 162 | 2.03 | **.044** |
|  |  | V-VI | 0.0259 | 0.0154 | 162 | 1.69 | .094 |
| A4 (N=172; 86F/86M) | Female | I | 0.0255 | 0.00934 | 79 | 2.73 | **.008** |
|  |  | III-IV | 0.0120 | 0.00742 | 79 | 1.61 | .111 |
|  |  | V-VI | 0.0111 | 0.00733 | 79 | 1.52 | .132 |
|  | Male | I | 0.00375 | 0.0318 | 79 | 0.12 | .906 |
|  |  | III-IV | -0.00793 | 0.0137 | 79 | -0.58 | .565 |
|  |  | V-VI | 0.00183 | 0.0105 | 79 | 0.17 | .862 |

Note. β = unstandardized pathway-specific PRS x amyloid chronicity interaction coefficient from propensity-score-matched, sex-stratified Huber-weighted robust regression, adjusted for APOE-ε4 status, age, and education. N reflects the full matched cohort; analytic N in individual models may be marginally lower due to listwise deletion of missing covariate data (see df). Reported p-values are nominal (uncorrected), consistent with the exploratory status of sex-stratified analyses in the main Statistical Analysis.

**Table S6.** Main effect of transport pPRS on amyloid burden, independent of tau-PET: Cross-sectional.

| **Cohort** | **Pathway** | **Amyloid measure** | **β** | **SE** | **df** | **t** | **p** |
| --- | --- | --- | --- | --- | --- | --- | --- |
| ADNI (N=441) | Transport | Amyloid chronicity | 3.39 | 3.84 | 435 | 0.88 | .379 |
|  |  | Centiloid | 10.4 | 14.9 | 435 | 0.70 | .487 |
| A4 (N=250) | Transport | Amyloid chronicity | -1.09 | 3.40 | 242 | -0.32 | .748 |
|  |  | Centiloid | -3.67 | 14.1 | 242 | -0.26 | .794 |

*Note.* β = unstandardized pathway-specific PRS coefficient from Huber-weighted robust regression predicting amyloid burden (amyloid chronicity or Centiloid) directly, adjusted for age, sex, education, and APOE-ε4 status. N reflects the full cross-sectional cohort; analytic N in individual models may be marginally lower due to listwise deletion of missing covariate data (see df). This analysis tests whether each pathway-specific PRS is independently associated with amyloid burden, as distinct from its interaction with amyloid chronicity on tau-PET deposition reported in Tables S3 and S9. No association reached statistical significance for any pathway, in either cohort or amyloid measure, indicating that pathway-specific PRS do not measurably predict amyloid burden itself, consistent with the interpretation that the pPRS x amyloid interactions reported for the transport pathway reflect genuine effect modification of the amyloid-tau relationship rather than a spurious association arising from PRS independently shaping amyloid accumulation.

**Table S7.** Direct main-effect association between transport pPRS and tau-PET deposition: Cross-sectional.

| **Cohort** | **Braak region** | **β** | **SE** | **df** | **t** | **p** |
| --- | --- | --- | --- | --- | --- | --- |
| ADNI (N=441) | I | −0.0214 | 0.0871 | 434 | −0.246 | .806 |
|  | III-IV | 0.0268 | 0.0588 | 434 | 0.455 | .649 |
|  | V-VI | 0.0251 | 0.0495 | 434 | 0.507 | .612 |
| A4 (N=250) | I | −0.0818 | 0.0728 | 241 | −1.12 | .262 |
|  | III-IV | −0.0757 | 0.0378 | 241 | −2.00 | .**047** |
|  | V-VI | −0.0631 | 0.0359 | 241 | −1.76 | .080 |

### *Note.* β = unstandardized transport pPRS main-effect coefficient from the direct-effect model (tau ~ PRS + age + sex + education + APOE-ε4 + amyloid chronicity), fit via Huber-weighted (M-estimation) robust regression on PC-residualized tau-PET Braak-stage values. N reflects the full cross-sectional cohort; analytic N in individual models may be marginally lower due to listwise deletion of missing covariate data (see df). The model contains no PRS x amyloid-chronicity interaction term, so β reflects the transport pPRS–tau association averaged across the amyloid-chronicity range, adjusting for the listed covariates.

### **Table S8**. Association between amyloid chronicity and tau-PET deposition in cross-sectional and longitudinal analyses of tau-PET in different Braak-stages

| **Cohort** | **Timepoint** | **Region** | **β** | **SE** | **p** |
| --- | --- | --- | --- | --- | --- |
| ADNI | Cross-sectional | Braak I | 0.015 | 0.001 | <.001 |
|  |  | Braak III-IV | 0.008 | 0.001 | <.001 |
|  |  | Braak V-VI | 0.005 | 0.001 | <.001 |
|  | Longitudinal | Braak I | 0.001 | <0.001 | <.001 |
|  |  | Braak III-IV | 0.001 | <0.001 | <.001 |
|  |  | Braak V-VI | 0.001 | <0.001 | <.001 |
| A4 | Cross-sectional | Braak I | 0.008 | 0.001 | <.001 |
|  |  | Braak III-IV | 0.004 | 0.001 | <.001 |
|  |  | Braak V-VI | 0.002 | 0.001 | <.001 |
|  | Longitudinal | Braak I | <0.001 | <0.001 | .241 |
|  |  | Braak III-IV | 0.001 | <0.001 | .002 |
|  |  | Braak V-VI | <0.001 | <0.001 | .020 |

*Note*. Robust linear regression (Huber M-estimation); cross-sectional outcome = residualized regional tau-PET SUVR at baseline (tau Braak PC residualised); longitudinal outcome = residualized annualized rate of change in regional tau-PET SUVR (tau_ROC, Braak PC residualised). All models covaried for age, sex, and education and APOE-ε4 status. No pathway PRS terms or interaction terms are included in this table, it reports the simple direct effect of amyloid chronicity (amyloid_chron) on tau, independent of and prior to any PRS moderation analysis. β = unstandardized coefficient; SE = standard error; p = two-sided p-value (uncorrected for multiple comparisons).

**Table S9.** Centiloid-based sensitivity analysis: transport pPRS x amyloid burden interaction on tau-PET Braak-stage deposition, ADNI and A4.

| **Cohort** | **Timepoint** | **Braak region** | **β** | **SE** | **df** | **t** | **p** | **p_FDR_BH** |
| --- | --- | --- | --- | --- | --- | --- | --- | --- |
| **ADNI** | Cross-sectional | I | 0.0042 | 0.0022 | 432 | 1.86 | .064 | .257 |
|  |  | III-IV | 0.0044 | 0.0015 | 432 | 2.89 | **.004** | **.024** |
|  |  | V-VI | 0.0040 | 0.0013 | 432 | 3.06 | **.002** | **.024** |
|  | Longitudinal | I | 0.00041 | 0.00022 | 202 | 1.81 | .072 | .207 |
|  |  | III-IV | 0.00097 | 0.00033 | 202 | 2.95 | **.0036** | **.021** |
|  |  | V-VI | 0.00107 | 0.00028 | 202 | 3.83 | **<.001** | **.002** |
| **A4** | Cross-sectional | I | 0.0045 | 0.0021 | 239 | 2.15 | **.033** | .098 |
|  |  | III-IV | 0.0018 | 0.0011 | 239 | 1.61 | .109 | .109 |
|  |  | V-VI | 0.0018 | 0.0011 | 239 | 1.67 | .097 | .109 |
|  | Longitudinal | I | 0.00046 | 0.00026 | 104 | 1.80 | .074 | .112 |
|  |  | III-IV | 0.00059 | 0.00032 | 104 | 1.83 | .070 | .112 |
|  |  | V-VI | 0.00031 | 0.00028 | 104 | 1.10 | .274 | .274 |

*Note.* β = unstandardized transport pPRS x Centiloid interaction coefficient from Huber-weighted robust regression, fit on tau-PET Braak-stage values residualized against the first five genomic principal components (PC1–PC5), adjusted for Centiloid x APOE-ε4 interaction, age, sex, and education. N reflects the full cohort; analytic N in individual models may be marginally lower due to listwise deletion of missing covariate data (see df). p_FDR_BH = Benjamini-Hochberg-corrected p-value; ADNI corrected across all four pathway-specific PRS x three Braak regions per timepoint (12-test set); A4 corrected across the three Braak regions for the transport pathway only (3-test set).

**Table S10.** Transport pathway PRS x amyloid-chronicity association with tau-PET: APOE-inclusive score

|  |  |  |  | **APOE-inclusive score** | | | |
| --- | --- | --- | --- | --- | --- | --- | --- |
|  | **Cohort:** | **Timepoint:** | **Braak region** | **β** | **t** | **p** | **FDR- p** |
|  | **ADNI** | Cross-sectional | Braak I | 0.0258 | 4.14 | **<.001** | **<.001** |
|  |  |  | Braak III/IV | 0.0207 | 4.91 | **<.001** | **<.001** |
|  |  |  | Braak V/VI | 0.0177 | 4.78 | **<.001** | **<.001** |
|  |  | Longitudinal | Braak I | 0.0009 | 1.56 | .120 | .228 |
|  |  |  | Braak III/IV | 0.0022 | 2.71 | **.007** | .065 |
|  |  |  | Braak V/VI | 0.0018 | 2.56 | **.011** | .065 |
| **A4** | | Cross-sectional | Braak I | 0.0026 | 0.28 | .779 | .779 |
|  | |  | Braak III/IV | 0.0101 | 2.13 | **.035** | .052 |
|  | |  | Braak V/VI | 0.0099 | 2.22 | **.027** | .052 |
|  | | Longitudinal | Braak I | 0.0010 | 1.13 | .263 | .341 |
|  | |  | Braak III/IV | 0.0015 | 1.39 | .167 | .341 |
|  | |  | Braak V/VI | 0.0009 | 0.96 | .341 | .341 |

*Note.* APOE-locus-exclude model (PRS x amyloid chronicity interaction, adjusted for age, sex, and education.. N reflects the full cohort; analytic N in individual models may be marginally lower due to listwise deletion of missing covariate data (see df). t = β / SE. p-values are for the PRS x amyloid-chronicity interaction term. For ADNI, FDR-corrected p is Benjamini-Hochberg-corrected within the pre-specified 12-test set (4 pathways × 3 Braak regions) per cohort/timepoint/arm. For A4, FDR-corrected p is Benjamini-Hochberg-corrected within a 3-test set (3 Braak regions x transport pathway only) per timepoint/arm, reflecting A4's role as a confirmatory validation of the pathway identified in the ADNI discovery analysis rather than an independent 4-pathway screen. These two correction scopes are not directly comparable across cohorts.
